# Magnesium‐Rich Mineral Water Improves Stool Consistency and Bowel Habits in Healthy Subjects: A Randomized Controlled Trial

**DOI:** 10.1111/nmo.70378

**Published:** 2026-06-12

**Authors:** Hideki Yoneda, Toshihiko Tomita, Takayuki Kitano, Daisuke Morishita, Mio Kodani, Norio Nishii, Hiroo Sei, Hirotsugu Eda, Toshiyuki Sato, Mikio Kawai, Yoko Yokoyama, Masataka Igeta, Takuya Okugawa, Hirokazu Fukui, Shinichiro Shinzaki

**Affiliations:** ^1^ Department of Gastroenterology Hyogo Medical University School of Medicine Nishinomiya Hyogo Japan; ^2^ Department of Medical Health Science Hyogo Medical University School of Medicine Nishinomiya Hyogo Japan; ^3^ Department of Biostatistics Hyogo Medical University School of Medicine Nishinomiya Hyogo Japan

**Keywords:** bowel movements, Bristol stool form scale, healthy subjects, magnesium, mineral water, randomized controlled trial

## Abstract

**Introduction:**

Magnesium‐rich mineral water influences stool consistency and bowel habits. However, randomized controlled trials directly comparing magnesium‐rich mineral water with soft water using standardized bowel outcomes remain limited.

**Methods:**

This single‐center, randomized, parallel‐group trial enrolled 40 healthy subjects who did not meet Rome IV criteria for chronic constipation or irritable bowel syndrome. After a 7‐day observation period, participants consumed either soft water or magnesium‐rich mineral water (1 L/day) for 14 days. The primary endpoint was change in weekly mean Bristol Stool Form Scale (BSFS) score from baseline to intervention week 2, analyzed using analysis of covariance. Secondary endpoints included spontaneous bowel movements (SBM; bowel movements without rescue medication), complete spontaneous bowel movements (CSBM; SBM with a sensation of complete evacuation), subjective global improvement, time to first SBM, and daily total fluid intake.

**Key Results:**

All participants completed the trial. Compared with soft water, magnesium‐rich mineral water significantly increased BSFS score at week 1 and week 2 (both *p* < 0.05). Weekly frequencies of SBM and CSBM were higher in the magnesium‐rich mineral water group. Subjective improvement at week 2 was more frequent with magnesium‐rich mineral water. Compared with the observation period, no difference in water intake was observed between the two groups.

**Conclusions and Inferences:**

In healthy subjects, magnesium‐rich mineral water was associated with stool softening and increased bowel movement frequency. No safety concerns were observed during the study period. These findings support further investigation of magnesium‐rich mineral water as a strategy for modulating bowel habits.

## Key Points

The effects of drinking water type on bowel habits have not been well established in randomized trials. This randomized controlled trial showed that magnesium‐rich mineral water significantly softened stool and increased bowel movement frequency compared with soft water. The mineral composition of drinking water, rather than fluid volume alone, may play an important role in bowel regulation.

## Plain Language Summary

Around the world, people drink many different types of water that vary in mineral content. Drinking water can influence bowel habits not only by the amount consumed, but also by its mineral content. In this study, we compared the effects of soft water and magnesium‐rich mineral water in healthy adults. Before the intervention, participants recorded their usual bowel habits during a one‐week baseline period under normal daily living conditions. After this baseline period, participants consumed one type of water daily for 2 weeks, and changes in bowel habits were compared with baseline.

We found that magnesium‐rich mineral water was associated with softened stools and an increased frequency of bowel movements compared with soft water. Participants who consumed magnesium‐rich mineral water were also more likely to report improvements in bowel movements. No safety concerns were observed during the study period.

These results suggest that the mineral content of drinking water, rather than volume alone, may play an important role in bowel function. Although this study was conducted in healthy individuals, magnesium‐rich mineral water may help prevent or alleviate constipation. Further studies in people with constipation are needed.

## Graphical abstract

In this single‐center, randomized, parallel‐group trial involving healthy adults, we demonstrate that magnesium‐rich mineral water significantly improved stool consistency, increased spontaneous and complete spontaneous bowel movements, and enhanced subjective bowel habit improvement compared with soft water without serious adverse events.

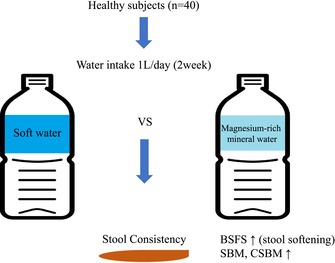

## 1 Introduction

The mineral composition of drinking water varies substantially across regions, particularly with respect to calcium and magnesium concentrations. Water hardness, a commonly used indicator of mineral content, is primarily determined by the concentration of these divalent cations [1]. In Japan, tap water is predominantly soft, whereas hard water is commonly consumed in many Western countries [2]. Although water hardness has been associated with several health outcomes, clinical evidence regarding its effects on stool consistency and bowel function remains limited, especially from randomized controlled trials [3, 4, 5].

Magnesium‐rich mineral water may promote bowel movements through an osmotic effect within the intestinal lumen, increasing stool water content and facilitating intestinal transit [6, 7, 8]. In Japan, magnesium‐based agents, most notably magnesium oxide, are widely prescribed as first‐line pharmacological therapy for chronic constipation, supporting the physiological plausibility of magnesium intake via drinking water as a non‐pharmacological approach to bowel regulation [9].

To date, observational studies and small interventional trials have suggested that consumption of hard water may be associated with improved bowel habits [10, 11, 12]. However, heterogeneity in outcome measures and methodological limitations have prevented definitive conclusions regarding efficacy and safety. In particular, randomized controlled trials comparing magnesium‐rich mineral water with soft water using internationally standardized bowel outcomes are scarce.

Accordingly, we conducted a randomized, parallel‐group trial to compare the effects and safety of soft water versus magnesium‐rich mineral water on stool consistency and bowel movement parameters in healthy subjects.

## 2 Materials and Methods

### 2.1 Study Design

This single‐center, randomized, parallel‐group trial was conducted between July and December 2025. Forty healthy subjects were enrolled and randomized 1:1 to receive either soft water (*n* = 20) or magnesium‐rich mineral water (*n* = 20).

Randomization was performed by an independent third party not otherwise involved in the study, using a computer‐generated permuted block randomization method (block size of five) stratified by sex. Allocation was implemented based on sequentially numbered participant identification codes. Commercially available bottled waters were used for both groups; all labels were removed, and the bottles were repackaged in plain cardboard boxes to minimize visual identification of the water type. However, complete blinding of participants could not be fully ensured because differences in taste may have allowed participants to infer the type of water consumed. Allocation information was securely managed by the principal investigator and was not disclosed during the study period except in emergency situations.

**FIGURE 1:**
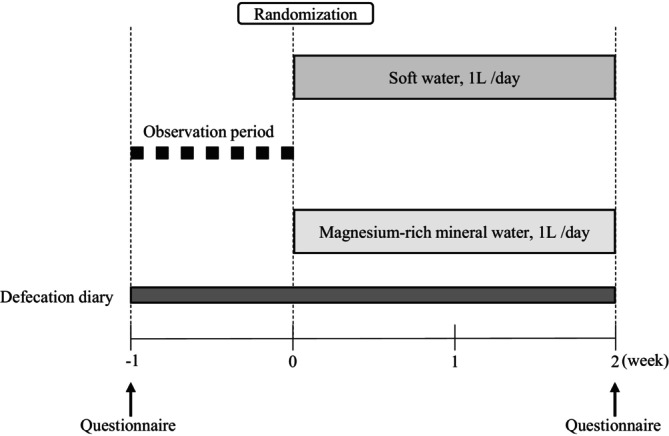
Study design: Overview of the 7‐day observation period and the 14‐day intervention period. Participants were instructed to consume 1 L/day of the assigned water.

Participants underwent a 7‐day observation period prior to water intake, followed by a 14‐day intervention period. During the intervention period, participants were instructed to consume 1 L per day of the assigned water. The soft water contained magnesium at a concentration of 0.15 mg/L with a total hardness of 27 mg/L, whereas the magnesium‐rich mineral water contained magnesium at 252 mg/L with a total hardness of 1033 mg/L. Participants in both groups were instructed not to consume more than 500 mL at a single time point and were encouraged to distribute intake evenly throughout the day. No restrictions were imposed on other lifestyle factors during the intervention period, including diet or additional fluid intake. Participants were instructed to maintain their habitual diet throughout the study; however, dietary intake, including fiber and total caloric intake, was not formally assessed. An overview of the study design is shown in Figure 1.

Throughout the 21‐day study period, participants recorded daily bowel movements, stool characteristics, abdominal symptoms, and fluid intake using a self‐administered diary developed by the investigators. Criteria for study discontinuation included vomiting, bradycardia, muscle weakness, somnolence, diarrhea occurring more than five times per day, or absence of bowel movements for more than three consecutive days. If any of these conditions occurred, water intake was discontinued and participants were instructed to seek medical evaluation; rescue treatment was permitted at the discretion of the investigators. When participants used rescue medication, bowel movement data for the day of use and the subsequent day were excluded from the analysis. In the soft water group, one participant used magnesium oxide twice as rescue medication on days 6 and 7 of the intervention period. Bowel movement data on the days of rescue medication use and the following day were excluded from the analysis. In the presence of abdominal symptoms, participants were allowed to adjust daily water intake within a range of 500–1000 mL. Although the type of rescue treatment was not prespecified, its use and details were recorded in the diary and reviewed during analysis.

This investigator‐initiated study received no commercial funding. All drinking water and packaging materials were purchased using institutional research funds, and shipment was handled by research staff not involved in data collection or analysis. Participants received a stipend of 20 USD as compensation for study participation.

The study was designed as an exploratory trial, and no formal sample size calculation was performed due to the limited availability of directly comparable prior randomized evidence. Based on feasibility considerations, a target sample size of 20 participants per group (total *n* = 40) was established. To provide quantitative context for this choice, we referred to the randomized placebo‐controlled parallel‐group trial of Mori et al. [13], which reported standard deviations for BSFS change of 1.15 and 1.03 in the active‐treatment and placebo groups, respectively. Although that study enrolled patients with chronic constipation rather than healthy individuals and reported mean BSFS change over a 4‐week treatment period, rather than the change in weekly mean BSFS score from baseline to intervention week 2, a study with 20 participants per group would correspond to an approximate two‐sided 95% confidence interval half‐width of about 0.68 points for the between‐group difference in the primary endpoint. Therefore, this sample size was considered acceptable for an exploratory precision‐based assessment of the treatment effect, although insufficient for a definitive hypothesis‐testing trial. Given the absence of an established minimal clinically important difference for the BSFS, the observed magnitude of change, although modest, may be considered clinically and physiologically plausible. The study protocol was approved by the Ethics Committee of Hyogo Medical University (approval number: 5048), and the trial was registered with the University Hospital Medical Information Network (trial registration: UMIN000060300). Written informed consent was obtained from all participants before enrollment. The study was conducted in accordance with the principles of the Declaration of Helsinki.

### 2.2 Participants

Enrolled in this study were 40 healthy subjects, defined as individuals who did not meet the Rome IV diagnostic criteria for chronic constipation or irritable bowel syndrome [14]. Prior to participation, all subjects received a full explanation of the study protocol and provided written informed consent.

The exclusion criteria were as follows: (1) age < 20 years or > 60 years; (2) current prescription of laxatives by a medical institution; (3) near‐daily use of over‐the‐counter laxatives; (4) intermittent use of over‐the‐counter laxatives with inability to discontinue use for at least 14 days prior to study initiation; (5) presence of renal dysfunction [15]; (6) presence of cardiac dysfunction; (7) history or diagnosis of hypermagnesemia; (8) history or diagnosis of hypokalemia; (9) habitual consumption of commercially available mineral water with high hardness that could not be discontinued after informed consent; (10) pregnancy or suspected pregnancy; (11) presence of serious systemic diseases, including malignancy or hematological disorders; and (12) history of aspiration pneumonia or clinically significant impairment of swallowing or mastication.

### 2.3 Outcomes

The primary outcome was the change in stool consistency, defined as the difference between the weekly mean Bristol Stool Form Scale (BSFS) score during the 1‐week observation period and the weekly mean BSFS score during the second week of the intervention period, and was compared between the soft water and magnesium‐rich mineral water groups [16]. Bowel habits were recorded daily using a self‐administered diary, and BSFS scores were summarized as weekly means for analysis.

Secondary outcomes included changes in bowel movement frequency, assessed by spontaneous bowel movements (SBM) and complete spontaneous bowel movements (CSBM) [17, 18]. For each outcome, the change in weekly mean frequency from the observation period to each week of the intervention period was calculated and compared between groups.

Responders for SBM and CSBM were defined as participants who showed an increase in bowel movement frequency compared with the observation period in both weeks of the intervention period and who achieved a weekly mean frequency of at least three bowel movements [19]. Subjective improvement in bowel habits was also assessed. Participants rated overall improvement weekly using a 5‐point Likert scale (1 = markedly improved, 2 = improved, 3 = slightly improved, 4 = unchanged, 5 = worsened), and those selecting a score of 1 or 2 at week 2 of the intervention period were classified as having symptomatic improvement [18, 20, 21].

In addition, the time to first SBM after initiation of water intake was analyzed as an exploratory outcome.

### 2.4 Questionnaires (Diary and Symptom Assessment)

Bowel habits were assessed using a self‐administered daily diary developed by the investigators. Participants recorded the daily frequency of SBM and CSBM, stool consistency as assessed by BSFS score, subjective improvement in bowel symptoms, and daily fluid intake. BSFS scores were summarized as weekly mean values for analysis.

Subjective improvement in bowel habits was assessed using a 5‐point Likert scale (1 = markedly improved, 2 = improved, 3 = slightly improved, 4 = unchanged, 5 = worsened). Participants who selected a score of 1 or 2 were classified as having symptomatic improvement.

Participants also recorded the volume of assigned drinking water consumed and total daily fluid intake throughout the study period. All diary‐derived variables were compared between the soft water and magnesium‐rich mineral water groups.

### 2.5 Statistical Analysis

The primary efficacy analysis population was the modified full analysis set (mFAS). Participants who had at least 1 day during the intervention period in which consumption of the assigned drinking water was < 500 mL were excluded from the mFAS according to prespecified criteria. No participants were excluded. There were no missing data in this study.

For the primary outcome, changes in stool consistency assessed by BSFS scores were analyzed using analysis of covariance (ANCOVA). The model included water type (soft water vs. magnesium‐rich mineral water) and sex as fixed factors, with the weekly mean BSFS score during the observation period included as a covariate. The dependent variable was defined as the change in weekly mean BSFS score from the observation period to week 2 of the intervention period. A two‐sided significance level of 5.0% was applied for the primary comparison.

For secondary outcomes, changes in the weekly mean frequency of SBM and CSBM at each intervention week were analyzed using similar ANCOVA models. In these analyses, water type and sex were included as fixed factors, and the corresponding weekly mean SBM or CSBM frequency during the observation period was included as a covariate. The dependent variables were defined as the changes in weekly mean SBM or CSBM frequency from the observation period to each week of the intervention period.

Responder rates for SBM and CSBM were compared between groups using logistic regression models, with water type included as the explanatory variable. Subjective improvement in bowel habits was similarly analyzed using logistic regression, defining symptomatic improvement as a score of 1 or 2 on the 5‐point Likert scale at week 2 of the intervention period.

Time to first SBM after initiation of water intake was summarized descriptively for each group. Cumulative incidence curves were generated using the Kaplan–Meier method, median survival times were estimated, and group differences were assessed using a stratified log‐rank test with sex as the stratification factor. In addition, hazard ratios were estimated using a Cox proportional hazards regression model.

Changes in daily fluid intake were analyzed using ANCOVA, with water type and sex included as fixed factors and the mean daily fluid intake during the observation period included as a covariate. The dependent variable was defined as the change in mean daily fluid intake from the observation period to each week of the intervention period.

Between‐group differences were presented as least squares means with 95% confidence intervals (CI). All statistical analyses were performed using JMP for Windows (JMP Student Edition version 18.2.2; SAS Institute Inc., Cary, NC, USA). Statistical significance was defined as a two‐sided *p* value < 0.05. No adjustment for multiple comparisons was performed.

## 3 Results

### 3.1 Participants and Baseline Characteristics

**FIGURE 2:**
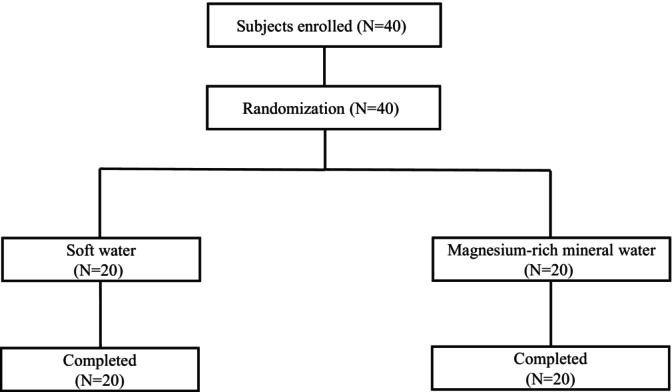
Disposition of study participants CONSORT‐style flow diagram showing enrollment, randomization, follow‐up, and analysis.

A total of 40 healthy subjects were enrolled and randomly assigned to the soft water group (*n* = 20) or the magnesium‐rich mineral water group (*n* = 20). No participants discontinued the study during either the observation or intervention periods, and all 40 participants were included in the analysis. The mean age of the study population was 38.9 ± 9.9 years, and the sex distribution was balanced (20 men and 20 women). The flow diagram of participant enrollment and allocation is shown in Figure 2.

Baseline characteristics are summarized in Table 1. There were no significant differences between the two groups with respect to age, sex, body mass index, smoking status, or alcohol consumption. In addition, no significant between‐group differences were observed during the observation period in terms of mean total daily fluid intake, weekly SBM, BSFS score, or CSBM. No serious adverse events related to the study intervention were reported in either group throughout the study period.

**TABLE 1:** Characteristics and symptoms at baseline of the participants.

	Soft water (*n* = 20)	Magnesium‐rich mineral water (*n* = 20)	Total
Age, years, mean ± SD	38.7 ± 9.9	39.0 ± 10.3	38.9 ± 9.9
Sex, *n* (% female)	10 (50)	10 (50)	20 (50)
BMI (kg/m^2^), mean ± SD	21.6 ± 2.9	21.9 ± 2.7	21.8 ± 2.8
Smoking, *n* (%)	3 (15)	3 (15)	6 (15)
Drinking, *n* (%)	7 (35)	4 (20)	11 (28)
Total daily fluid intake (mL/day), mean ± SD	1603 ± 589	1532 ± 873	1567 ± 736
SBM/week, mean ± SD	8.4 ± 3.9	7.7 ± 2.7	8.0 ± 3.3
Bristol Stool Form Scale, mean ± SD	3.9 ± 0.7	3.8 ± 0.6	3.8 ± 0.6
CSBM/week, mean ± SD	6.9 ± 4.7	6.8 ± 2.7	6.8 ± 4.0

Abbreviations: CSBM, Complete spontaneous bowel movement; SBM, Spontaneous bowel movement; SD, standard deviation.

During the intervention period, 20% of participants (8/40) continued the study with a reduced intake of the assigned drinking water; however, all of these participants consumed at least 500 mL/day. This proportion was 25% (5/20) in the magnesium‐rich mineral water group and 15% (3/20) in the soft water group. The actual intake of the assigned water was 946 mL/day (95% CI, 793 to 1099) in week 1 and 935 mL/day (95% CI, 782 to 1088) in week 2 in the soft water group, and 900 mL/day (95% CI, 716 to 1084) in week 1 and 892 mL/day (95% CI, 690 to 1094) in week 2 in the magnesium‐rich mineral water group. The total daily fluid intake was 1907 mL/day (95% CI, 1650 to 2165) in week 1 and 1862 mL/day (95% CI, 1608 to 2116) in week 2 in the soft water group, and 1927 mL/day (95% CI, 1529 to 2324) in week 1 and 1917 mL/day (95% CI, 1522 to 2312) in week 2 in the magnesium‐rich mineral water group.

### 3.2 Change in BSFS Score After Water Intake

At week 2 of the intervention period, the adjusted mean change in BSFS score from baseline was 0.79 (95% CI, 0.45 to 1.13) in the magnesium‐rich mineral water group and 0.22 (95% CI, −0.11 to 0.56) in the soft water group. ANCOVA demonstrated a significantly greater increase in BSFS score in the magnesium‐rich mineral water group compared with the soft water group, with a between‐group difference of 0.57 (95% CI, 0.09 to 1.04; *p* = 0.02).

**FIGURE 3:**
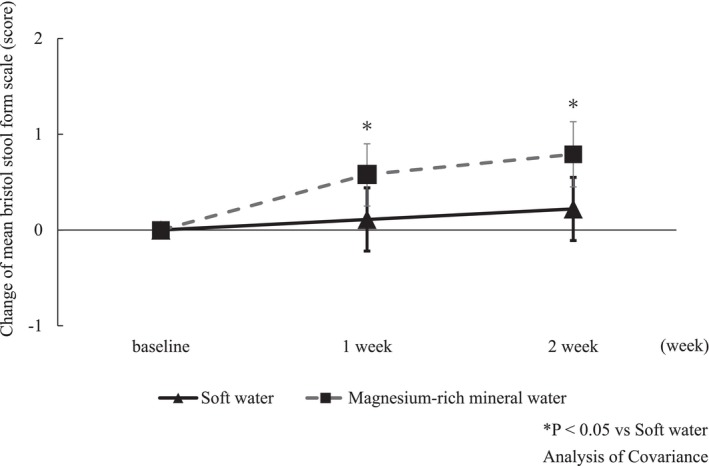
Changes in Bristol Stool Form Scale (BSFS) score after water intake Weekly mean BSFS scores and change from baseline (observation period). *p < 0.05 versus soft water. Data are presented as least‐squares means with 95% confidence intervals unless otherwise stated.

Similarly, at week 1 of the intervention period, the adjusted mean change in BSFS score from baseline was 0.58 (95% CI, 0.28 to 0.88) in the magnesium‐rich mineral water group and 0.15 (95% CI, −0.14 to 0.44) in the soft water group. The between‐group difference was 0.46 (95% CI, 0.007 to 0.92; *p* = 0.04), indicating significant stool softening in the magnesium‐rich mineral water group (Figure 3).

### 3.3 Change in SBM After Water Intake

At week 1 of the intervention period, the adjusted mean change in SBM from baseline was 2.5 movements per week (95% CI, 1.0 to 4.1) in the magnesium‐rich mineral water group and 0.05 movements per week (95% CI, −1.4 to 1.5) in the soft water group. ANCOVA demonstrated a significantly greater increase in SBM frequency in the magnesium‐rich mineral water group compared with the soft water group, with a between‐group difference of 2.5 movements per week (95% CI, 0.3 to 4.6; *p* = 0.02).

**FIGURE 4:**
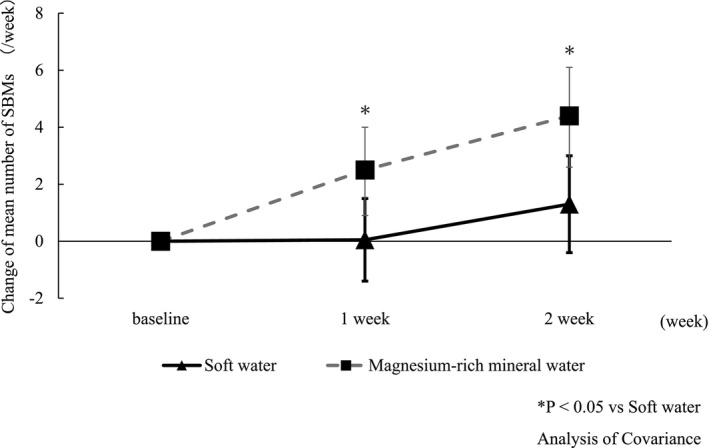
Changes in spontaneous bowel movements (SBM) after water intake: Weekly mean SBM values and change from baseline. *p < 0.05 versus soft water.

At week 2, the adjusted mean change in SBM further increased to 4.4 movements per week (95% CI, 2.7 to 6.2) in the magnesium‐rich mineral water group, whereas the change in the soft water group was 1.3 movements per week (95% CI, −0.4 to 3.0). The between‐group difference remained statistically significant, favoring the magnesium‐rich mineral water group, with a difference of 3.1 movements per week (95% CI, 0.6 to 5.6; *p* = 0.01) (Figure 4).

In contrast, the proportion of SBM responders was 65% in the magnesium‐rich mineral water group and 45% in the soft water group, with no statistically significant difference between the groups (*p* = 0.20).

### 3.4 Change in CSBM After Water Intake

At week 1 of the intervention period, the adjusted mean change in CSBM from baseline was 2.3 movements per week (95% CI, 0.6 to 4.0) in the magnesium‐rich mineral water group and −0.1 movements per week (95% CI, −1.8 to 1.5) in the soft water group. Analysis of covariance showed a significantly greater increase in CSBM frequency in the magnesium‐rich mineral water group compared with the soft water group, with a between‐group difference of 2.5 movements per week (95% CI, 0.1 to 4.9; *p* = 0.03).

**FIGURE 5:**
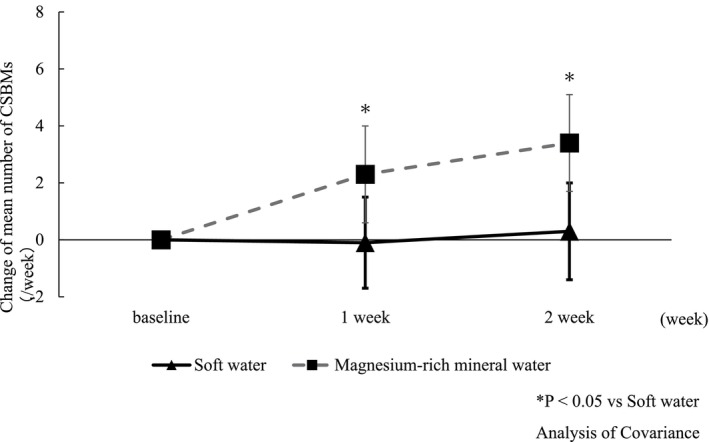
Changes in complete spontaneous bowel movements (CSBM) after water intake: Weekly mean CSBM values and change from baseline. *p < 0.05 versus soft water.

At week 2, the adjusted mean change in CSBM further increased to 3.4 movements per week (95% CI, 1.7 to 5.1) in the magnesium‐rich mineral water group, whereas the change in the soft water group was 0.3 movements per week (95% CI, −1.4 to 2.0). The between‐group difference remained statistically significant, favoring the magnesium‐rich mineral water group, with a difference of 3.1 movements per week (95% CI, 0.6 to 5.5; *p* = 0.01) (Figure 5).

In contrast, the proportion of CSBM responders was 60% in the magnesium‐rich mineral water group and 35% in the soft water group, with no statistically significant difference between the groups (*p* = 0.11).

### 3.5 Effect of Overall Symptomatic Improvement in Subjects

**FIGURE 6:**
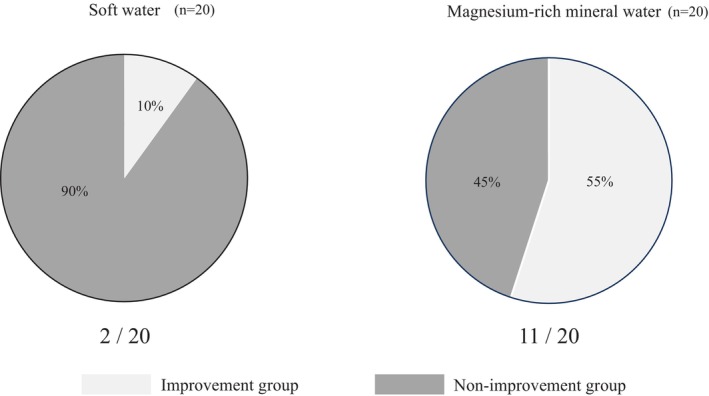
Overall symptomatic improvement at week 2: Proportion of participants reporting improvement (Likert scores of 1 or 2) at intervention week 2 in each group.

At week 2 of the intervention period, the proportion of subjects reporting overall improvement in bowel symptoms was significantly higher in the magnesium‐rich mineral water group than in the soft water group (55% vs. 10%, respectively; *p* = 0.005) (Figure 6).

### 3.6 Time to First SBM After Water Intake

The median time to the first SBM after initiation of the assigned intervention was 9.5 h (95% CI, 5.7 to 13.2) in the magnesium‐rich mineral water group and 11.2 h (95% CI, 6.9 to 15.5) in the soft water group.

**FIGURE 7:**
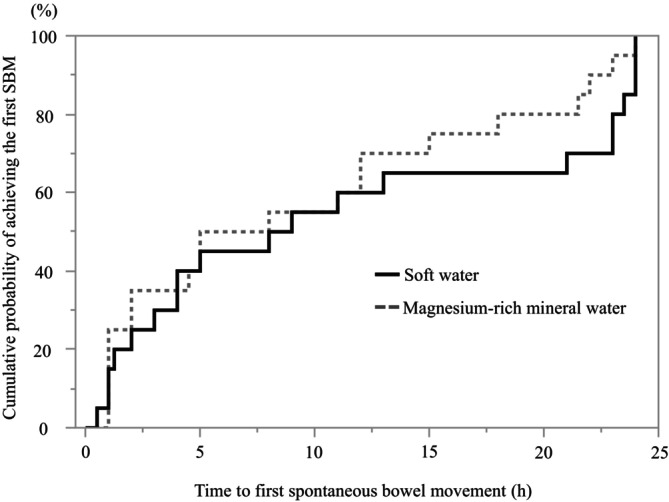
Time to first spontaneous bowel movement. Cumulative incidence curves of time to first spontaneous bowel movement, calculated as 1 minus the Kaplan–Meier estimate.

Kaplan–Meier analysis demonstrated no significant difference between the two groups. In the stratified Cox proportional hazards model, there was no significant difference in the hazard of the first bowel movement between the two groups (HR 1.31; 95% CI, 0.69 to 2.48; *p* = 0.40) (Figure 7).

### 3.7 Total Fluid Intake Between Study Groups

At week 1 of the intervention period, the adjusted mean change in daily total fluid intake from the observation period was 389 mL/day (95% CI, 239 to 540) in the magnesium‐rich mineral water group and 309 mL/day (95% CI, 159 to 460) in the soft water group. The between‐group comparison using analysis of covariance showed a between‐group difference of 79 mL/day (95% CI, −132 to 292; *p* = 0.45).

At week 2, the adjusted mean change in daily fluid intake was 379 mL/day (95% CI, 217 to 541) in the magnesium‐rich mineral water group and 265 mL/day (95% CI, 103 to 427) in the soft water group. The between‐group difference was 114 mL/day (95% CI, −114 to 343), which indicated no significant difference in daily fluid intake between the two groups (*p* = 0.31).

## 4 Discussion

This randomized parallel‐group controlled trial compared the effects of soft water and magnesium‐rich mineral water on stool characteristics and bowel function. Magnesium‐rich mineral water is widely consumed in Western countries. We found that compared with soft water intake, the intake of magnesium‐rich mineral water was associated with increases in BSFS scores, indicating stool softening, as well as increases in both SBM and CSBM. In addition, subjective improvement in bowel symptoms was more frequent in the magnesium‐rich mineral water group. The primary outcome showed a statistically significant between‐group difference in BSFS scores of approximately 0.57 points. However, the clinical relevance of this magnitude of change should be interpreted cautiously. To our knowledge, a minimal clinically important difference for the BSFS has not been clearly established. Given that the study population consisted of healthy individuals, a one‐point change in BSFS may not be easily achieved through water intake alone. Therefore, the observed magnitude of change, although modest, may be considered physiologically plausible and potentially relevant in this context. In contrast, no between‐group difference was observed in the time to the first spontaneous bowel movement. No difference in total daily fluid intake was observed between the two groups. Collectively, these findings suggest that not only the quantity of water intake but also its qualitative characteristics (specifically mineral composition) may influence bowel function.

Lifestyle modification, including increased dietary fiber intake, physical activity, adequate hydration, and bowel training, is recommended as first‐line management for constipation [22, 23, 24, 25]. However, clinical evidence evaluating the independent effects of water intake on bowel function remains limited, and studies focusing on differences in water type are particularly scarce [26, 27, 28]. A key strength of the present study is the comparison of water types under conditions of standardized water intake, which allows for assessment of the impact of mineral composition independent of fluid volume. Our findings provide novel evidence supporting the role of water quality in bowel regulation and contribute to the scientific basis for hydration guidance in clinical practice [29].

The observed improvements in stool consistency and bowel movement frequency in the magnesium‐rich mineral water group may be explained, at least in part, by the osmotic effects of magnesium. Magnesium is known to retain water within the intestinal lumen, increasing stool water content and facilitating stool passage [6, 7, 8]. In Japan, magnesium oxide is widely prescribed as a first‐line agent for chronic constipation, and its laxative effect is well established [9]. The magnesium content in the magnesium‐rich mineral water used in this study was approximately 252 mg/day (assuming an intake of 1 L/day). In contrast, magnesium oxide, a commonly used laxative for chronic constipation, is typically administered at a dose of approximately 1 g/day, corresponding to about 600 mg of elemental magnesium [30]. Therefore, the magnesium intake from mineral water in the present study was substantially lower than that typically used in pharmacological supplementation, indicating that the observed effects were achieved at a non‐pharmacological dose level.

Although total daily fluid intake increased during the intervention period in both groups, stool consistency softened (as reflected by higher BSFS scores) and both SBM and CSBM frequencies increased only in the magnesium‐rich mineral water group. This finding suggests that the beneficial effects on bowel function cannot be explained solely by increased fluid volume. Rather, the mineral composition of the water appears to play a crucial role. Recent studies have suggested that both mineral content and fluid intake may influence the gut microbiota, and accumulating evidence supports a link between constipation and alterations in gut microbial composition [31, 32, 33]. The present study did not assess gut microbiota or other biological markers. Therefore, the mechanisms underlying the observed improvements in bowel function cannot be determined in this study. It is possible that changes in the intestinal environment associated with mineral water intake contributed to these effects; however, such interpretations remain speculative and warrant further investigation.

Several previous studies have examined the relationship between hard water consumption and bowel function. Observational studies have suggested a lower prevalence of constipation among residents in hard water regions; however, these studies were limited by insufficient adjustment for confounding factors such as diet, physical activity, and lifestyle habits [34]. Small interventional studies have reported improvements in bowel movements following mineral water intake, but most were single‐arm trials or comparisons between different types of magnesium‐rich mineral water [10, 11, 12]. Randomized controlled trials using soft water as a comparator are extremely limited. Moreover, many previous studies relied solely on bowel movement frequency, without incorporating internationally standardized outcome measures such as the BSFS score or CSBM.

Compared with these earlier studies, the present study has several methodological strengths. It was conducted as a randomized parallel‐group controlled trial with soft water as a comparator, and bowel function was assessed using multiple validated outcomes, including the BSFS score, SBM, CSBM, and subjective symptom improvement. In addition, a baseline observation period was included to characterize habitual bowel patterns, and changes in fluid intake were statistically adjusted for in the analysis. These features enhance the robustness and interpretability of our findings. Nevertheless, certain limitations are shared with prior studies, including the restriction to healthy subjects and the relatively short intervention period. Larger and longer‐term studies involving patients with chronic constipation are therefore needed.

From a clinical perspective, the present findings suggest that habitual intake of magnesium‐rich mineral water may represent a non‐pharmacological option to improve bowel function. Such an approach may be particularly attractive for individuals who are reluctant to use medications or in whom lifestyle‐based interventions are preferred. Improvements in stool consistency and bowel movement frequency are closely linked to quality of life in patients with constipation, underscoring the potential clinical relevance of this strategy [35, 36, 37].

Several limitations of this study should be acknowledged. First, the study population consisted of healthy subjects, and the results cannot be directly extrapolated to patients with chronic constipation. Second, the sample size was relatively small, and the study was exploratory in nature. Third, the intervention period was limited to 2 weeks, precluding evaluation of long‐term effects and safety. Serum electrolytes were not routinely measured in this study; however, the magnesium intake from the intervention water was substantially lower than that typically used in pharmacological supplementation, and no clinical symptoms suggestive of hypermagnesemia were observed in these healthy participants. Fourth, lifestyle factors such as dietary intake and physical activity were not strictly controlled. Although participants were instructed to maintain their habitual diet during the study period, dietary adherence was not objectively verified, and dietary intake, including fiber intake, was not formally recorded. Therefore, residual confounding related to dietary variation cannot be excluded, and this should be considered when interpreting the findings. In addition, although water bottles were unlabeled, differences in taste may have allowed participants to infer group allocation, making complete blinding difficult. While the observed data do not allow a definitive assessment of the extent of these biases, expectation bias related to subjective outcomes—specifically, the expectation that magnesium‐rich mineral water would improve bowel symptoms—cannot be excluded. However, improvements were also observed in objective measures, including the BSFS score, SBM, and CSBM, suggesting that the findings are unlikely to be explained solely by subjective bias. Furthermore, missing data were not imbalanced between groups, and no evidence suggestive of reporting bias was observed. Finally, multiple secondary endpoints were analyzed without adjustment for multiplicity. Therefore, these findings should be interpreted as exploratory and hypothesis‐generating, and the possibility of a type I error cannot be excluded.

## 5 Conclusion

This exploratory randomized controlled trial demonstrated that intake of magnesium‐rich mineral water was associated with stool softening and increased bowel movement frequency in healthy subjects. No safety concerns were observed in healthy individuals during the study period. These findings suggest that the mineral composition of drinking water, rather than fluid volume alone, may play an important role in bowel regulation. Further studies in patients with chronic constipation and mechanistic investigations incorporating gut microbiota analyses are warranted to clarify the clinical utility of mineral water as a non‐pharmacological intervention.

## Author Contributions

Conceptualization, Hideki Yoneda and Toshihiko Tomita; methodology, Hideki Yoneda, Toshihiko Tomita, Takayuki Kitano, Daisuke Morishita, Mio Kodani, Norio Nishii, Hiroo Sei, Hirotsugu Eda, Toshiyuki Sato, Mikio Kawai, Yoko Yokoyama, Takuya Okugawa, and Hirokazu Fukui; validation, Hideki Yoneda and Toshihiko Tomita; formal analysis, Hideki Yoneda, Mikio Kawai, and Toshihiko Tomita; investigation, Hideki Yoneda, Toshihiko Tomita, Takayuki Kitano, Daisuke Morishita, Mio Kodani, Norio Nishii, Hiroo Sei, Hirotsugu Eda, Toshiyuki Sato, Mikio Kawai, Yoko Yokoyama, Takuya Okugawa, Hirokazu Fukui, and Shinichiro Shinzaki; resources, Toshihiko Tomita, Takuya Okugawa, and Shinichiro Shinzaki; data curation, Hideki Yoneda and Toshihiko Tomita; writing – original draft preparation, Hideki Yoneda and Toshihiko Tomita; writing – review and editing, Hideki Yoneda, Toshihiko Tomita, Hirokazu Fukui, and Shinichiro Shinzaki; supervision, Hirokazu Fukui and Shinichiro Shinzaki; project administration, Hideki Yoneda, Toshihiko Tomita, and Shinichiro Shinzaki. In particular, Takayuki Kitano, Daisuke Morishita, Mio Kodani, Norio Nishii, Hiroo Sei, Hirotsugu Eda, Toshiyuki Sato, Mio Kodani, Yoko Yokoyama, and Takuya Okugawa contributed to data collection, sample processing, and investigation. All authors have read and agreed to the published version of the manuscript.

## Funding

This investigator‐initiated study received no commercial funding.

## Conflicts of Interest

The authors declare no conflicts of interest.

## Data Availability

The datasets generated and/or analyzed during the current study are available from the corresponding author upon reasonable request.
